# Assessing gait, balance, and muscle strength among breast cancer survivors with chemotherapy-induced peripheral neuropathy (CIPN): study protocol for a randomized controlled clinical trial

**DOI:** 10.1186/s13063-022-06294-w

**Published:** 2022-04-27

**Authors:** Patricia Teran-Wodzinski, Douglas Haladay, Tuan Vu, Ming Ji, Jillian Coury, Alana Adams, Lauren Schwab, Constance Visovsky

**Affiliations:** 1 School of Physical Therapy & Rehabilitation Science, Morsani College of Medicine, University of South Florida, 12901 North Bruce B. Downs Blvd., MDC 077, Tampa, FL 33612-4766 USA; 2 Department of Neurology, University of South Florida, 12901 Bruce B. Downs Blvd., MDC55, Tampa, FL 33612 USA; 3 College of Nursing, University of South Florida, 12901 Bruce B. Downs Blvd., MDC Box 22, Tampa, FL 33612 USA

**Keywords:** Therapeutic exercise, Chemotherapy-induced peripheral neuropathy, Breast cancer

## Abstract

**Background:**

Chemotherapy-induced peripheral neuropathy (CIPN) is a common and understudied consequence of taxane chemotherapy for breast cancer treatment. CIPN symptoms include numbness combined with tingling sensations, persistent shooting, stabbing, or burning pain even in the absence of painful stimuli, lower extremity muscle weakness, and impaired balance. CIPN symptoms often persist for a long time after completion of chemotherapy, causing significant loss of functional abilities and increased risk of falls. Persistent CIPN caused by taxanes represents a therapeutic challenge due to the limited treatment options. Resistance exercise has shown promising results; however, the effect of exercise on CIPN remains understudied. This study aims to assess the effects of exercise on gait, balance, and lower extremity muscle strength after a 16-week home-based exercise program compared to an educational attention control condition.

**Methods:**

A sample of 312 women who completed taxane-based chemotherapy for breast cancer and have symptomatic neuropathy is recruited from a community-dwelling sample. Participants are randomized to either a 16-week Home-Based Physical Activity Intervention or an Educational Attention control group. The home-based intervention protocol consists of targeted lower extremity stretches, followed by 10 min each of gait/balance and 10 min of resistive training accessed by hyperlink or DVD. An Exercise Diary records quantitative exercise data. The gait assessment includes temporospatial parameters and lower extremity joint angles using APDM motion sensors. Participants’ balance is assessed using the Sensory Organization Test (SOT) performed using a NeuroCom Balance Master. Isometric strength of hip, knee, and ankle flexor and extensor muscles is assessed using an isokinetic dynamometer, Biodex BX Advantage. In addition, we assess neuropathy symptoms using the FACT-Taxane Additional Concerns Subscale and nerve conduction velocity of the sural and peroneal nerve action potentials. Outcomes are assessed at baseline (prior to randomization) and 16 weeks.

**Discussion:**

There are currently no evidence-based interventions that address the functional declines associated with CIPN. If successful, this program is simple and easy to implement in the standard of care for individuals with CIPN. Gait and balance training have the potential to reduce physical dysfunction associated with CIPN and reduce the burden of disease in cancer survivors.

**Trial registration:**

ClinicalTrials.gov NCT04621721. Registered on August 3, 2020. ClincialTrials.gov is a primary registry of the World Health Organization International Clinical Trials Registry Platform (WHO ICTEP) network and includes all items from the WHO Trial Registration data set in Trial registration.

**Supplementary Information:**

The online version contains supplementary material available at 10.1186/s13063-022-06294-w.

## Administrative information

**Table:** 

Title	Assessing gait, balance, and muscle strength among breast cancer survivors with chemotherapy-induced peripheral neuropathy (CIPN): study protocol for a randomized controlled clinical trial
Trial registration	NCT04621721 [ClinicalTrials.gov]. Registered on August 3, 2020, https://clinicaltrials.gov/ct2/show/NCT04621721 World Health Organization International Clinical Trials Registry Platform (WHO ICTEP) network: https://trialsearch.who.int/Trial2.aspx?TrialID=NCT04621721
Protocol version	Version #2 of 03-14-2022
Funding	National Cancer Institute, NIH: 1R01CA229681-01A1
Author details	P. Teran-Wodzinski: School of Physical Therapy & Rehabilitation Sciences, University of South Florida D. Haladay: School of Physical Therapy & Rehabilitation Sciences, University of South Florida T. Vu: Department of Neurology, University of South Florida M. Ji: College of Nursing, University of South Florida J. Coury: College of Nursing, University of South Florida A. Adams: School of Physical Therapy & Rehabilitation Sciences, University of South Florida L. Schwab: College of Nursing, University of South Florida C. Visovsky: College of Nursing, University of South Florida
Name and contact information for the trial sponsor	National Cancer Institute (NCI) Alexis Bakos, PhD, MPH, RN Program Director Supportive Care & Symptom Management Program Community Oncology & Prevention Trials Research Group Division of Cancer Prevention, National Cancer Institute National Institutes of Health 9609 Medical Center Dr., 5E438-MSC9785 Bethesda, MD 20892 301-921-5970 (office cellphone) Investigator initiated clinical trial C. Visovsky (Principal Investigator) cvisovsk@usf.edu
Role of sponsor	This is an investigator initiated clinical trial. Therefore, the sponsor played no role in the design of the study and collection, analysis, and interpretation of data and in writing the manuscript.

## Introduction

### Background and rationale

Approximately 13% of women in the USA will develop invasive breast cancer throughout their lifetime. In 2021, it was estimated that 281,550 new cases of invasive breast cancer are expected to be diagnosed in women in the USA. The treatment of invasive breast cancer will require a chemotherapy regimen that includes taxanes as a standard treatment for invasive breast cancer [1]. Taxane-based chemotherapy can result in chemotherapy-induced peripheral neuropathy (CIPN) [1, 2]. CIPN includes numbness, tingling sensations, persistent shooting, stabbing, burning pain or loss of cutaneous sensation, lower extremity muscle weakness, and impaired balance [3, 4]. These symptoms are distributed distal to proximal, with the lower extremities being affected first. Chemotherapy-induced peripheral neuropathy can persist long after completion of chemotherapy, causing significant loss of functional abilities, compromising the quality of life, and increasing the risk of falls [4, 5]. Taxanes affect thick myelinated nerve fibers, resulting in muscle strength and position sense. Motor and sensory neuronal loss in the lower extremities results in weakness of large lower extremity muscle groups and reduced gait performance and ability to compensate for changes in terrain [6, 7]. Hypotonia from lower peripheral motor neuron and muscle involvement can result in an unsteady gait [8]. Persistent CIPN caused by taxanes represents a therapeutic challenge due to the limited treatment options.

Studies in animal models and humans have suggested that resistance exercise and balance training may offer the possibility of reducing the effects of peripheral neuropathy. Studies in animal models have shown that a treadmill exercise program before taxane administration and continuing over weeks prevented the development of peripheral neuropathy [9]. In addition, treadmill exercise upregulated protective neurotrophic factors that may be responsible for the neuroprotection achieved with exercise [10]. Recent randomized control trials (RCT) have found that exercise may reduce CIPN symptoms, especially in women with breast cancer [11, 12], and may help cancer survivors regulate inflammation through endogenous cytokine pathways [13]. In studies of individuals with chronic peripheral nerve disorders, short-term (6 and 12 weeks) home and community-based exercise programs increased average muscle strength, improved walk time, and significant improvements in activity limitation and overall health [14, 15]. The effects of exercise on CIPN caused by taxanes are promising and remain understudied. This randomized clinical trial (RCT) aims to assess the effects of exercise on gait, balance, and lower extremity muscle strength in 312 women randomized to either a 16-week home-based gait/balance intervention or an attention control educational cancer survivorship condition.

## Methods/design

### Study design

A two-group, 16-week randomized clinical trial is designed to address persistent taxane-induced peripheral neuropathy in women treated for invasive breast cancer. The study design is a parallel, two-arm, randomized controlled trial with a 1:1 ratio between groups.

**Fig. 1:**
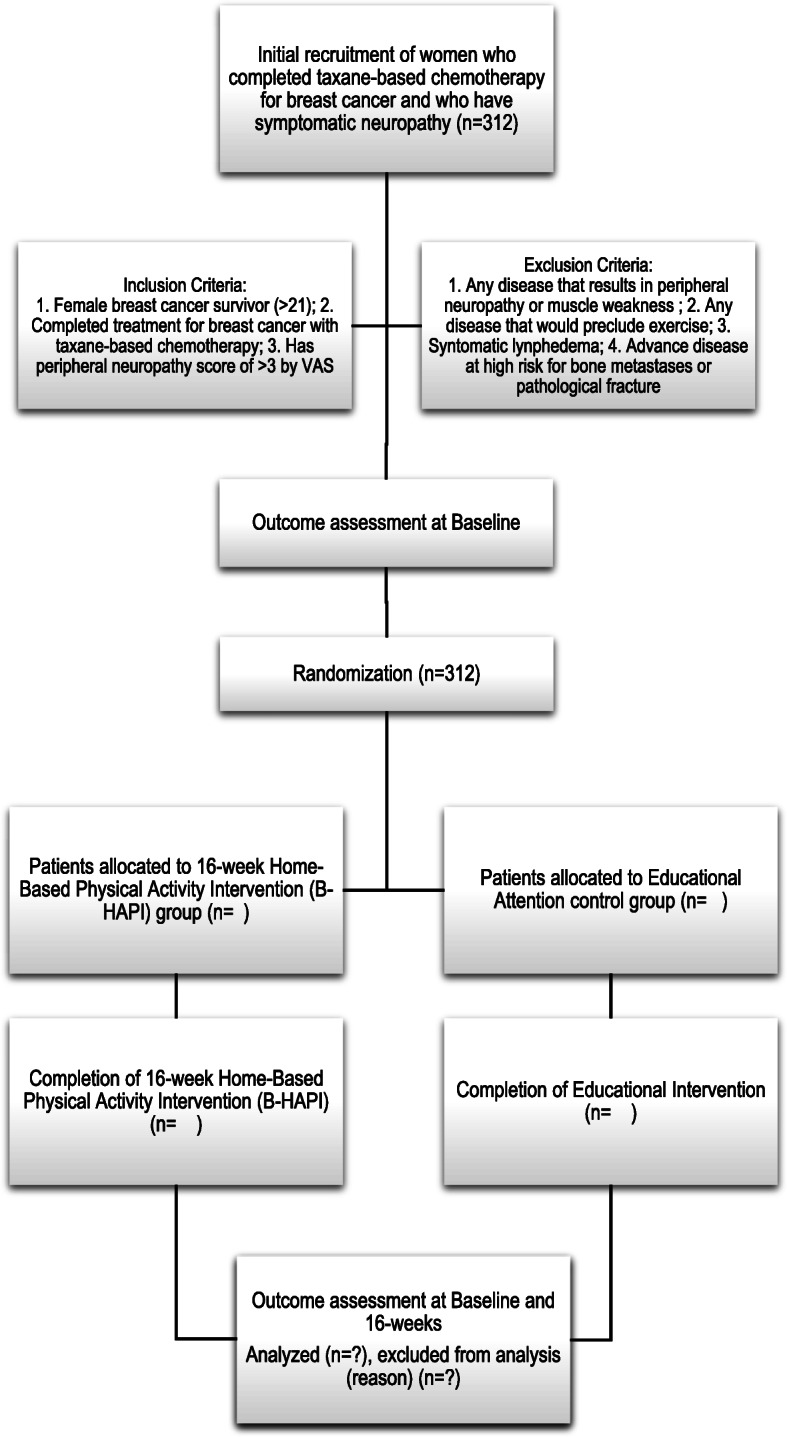
Participant flow diagram

**Fig. 2:**
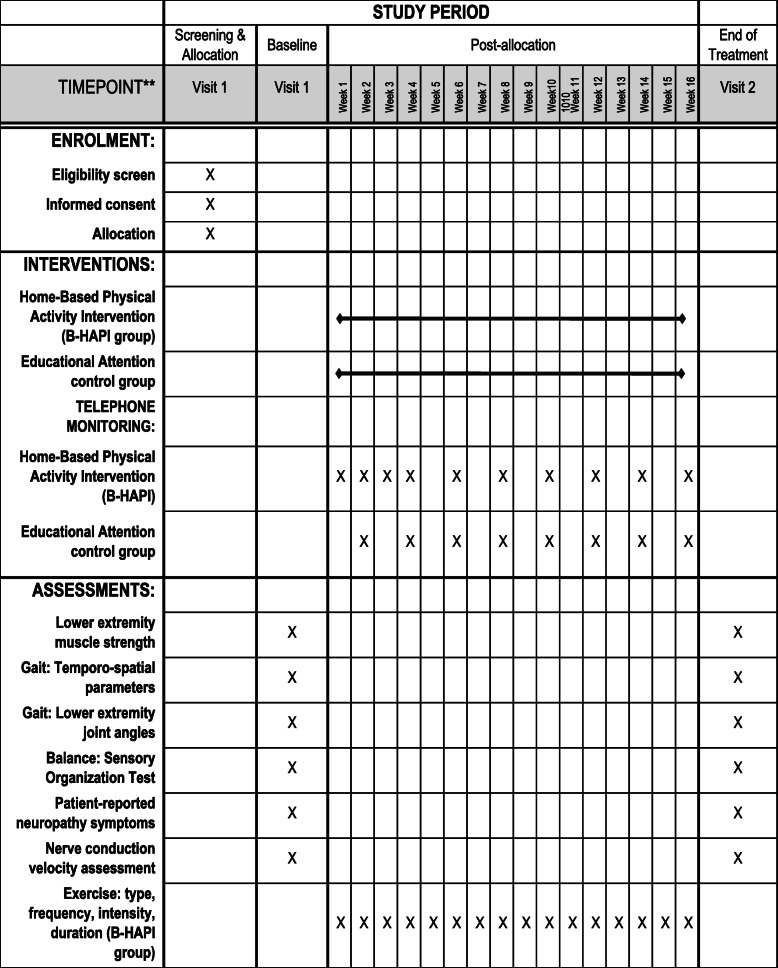
Schedule of enrolment, interventions, and assessments

A sample of 312 women who completed taxane-based chemotherapy for breast cancer and have symptomatic neuropathy (≥ 3 on a neuropathy visual analog scale VAS) ≥ 6 months following completion of taxane-based chemotherapy will be recruited. Participants are randomized to either a Home-Based Physical Activity Intervention (B-HAPI) for persistent taxane-induced neuropathy or an educational attention control group. Assessment of gait, balance, strength of lower extremity muscles, neuropathy symptoms, and nerve conduction velocity will be collected at baseline (prior to randomization) and 16 weeks. The participant flow diagram is shown in Fig. 1. The protocol follows the Standard Protocol Items: Recommendations for Interventional Trials (SPIRIT) Figure (Fig. 2) and Checklist (Additional File 1) [16].

### Study setting and recruitment

Assessment occurs at the Human Functional Performance Laboratory at the University of South Florida (HFPL). Community-dwelling breast cancer survivors are recruited from local breast cancer support groups, breast cancer clinics, local churches, and advertisements in local community papers. In addition, we use social media recruitment (multiple ads targeting different ages, race/ethnic groups), Facebook, Twitter, and Instagram. We distribute recruitment flyers at clinics, breast cancer support groups, and Hispanic and Black church outreach. Participants are considered for inclusion if they meet the criteria as defined below.

### Eligibility criteria

#### Primary inclusion criteria

Participants must meet the following criteria to be eligible for the study:

- Female breast cancer survivor (≥ 21 years old).
- Have completed treatment (≥ 6 months) for invasive breast cancer with taxane-based chemotherapy.
- Have a peripheral neuropathy score of ≥ 3 by VAS rating.

#### Primary exclusion criteria

If the participants meet any of the following criteria during screening, they are not eligible for the study:

- Have any disease (e.g., diabetes, human immunodeficiency virus) resulting in peripheral neuropathy or muscle weakness (chronic fatigue syndrome, multiple sclerosis, spinal cord tumors or injuries, stroke).
- Have any disease that would preclude exercise (preexisting cardiopulmonary disease, bone metastasis).
- Have symptomatic lymphedema or advanced disease at high risk for bone metastases and pathologic fracture.

#### Who will take the informed consent?

Participants who are 6 months or more post-treatment completion for non-metastatic breast cancer with taxane chemotherapy and who have a chemotherapy-induced peripheral neuropathy (CIPN) VAS score of ≥ 3 are screened for eligibility to participate in this study based on the criteria mentioned above. After the interested participant has been assessed as eligible, they are invited to the HFPL to discuss any remaining questions and sign the informed consent.

### Interventions

#### Intervention protocol

The Home-Based Physical Activity Intervention (B-HAPI) consists of a 16-week, home-based gait/balance training and progressive resistance exercises using resistance power bands for lower extremities. The exercise program contains detailed, easy-to-follow demonstrations for each gait/balance training and resistance exercise training led by a physical therapist. Each participant allocated to the intervention group receives an exercise plan, which is accessed via a link or DVD, demonstrating the correct performance of the exercises, and captures in a weekly Exercise Diary. In addition, all exercise sessions use an Exercise Diary to record quantitative exercise data. Intervention participants are instructed to perform the gait/balance and resistance exercises 3 days per week. The intervention group begins with a light warm-up and stretching activity at the beginning of the program, working into the 10 min each of gait/balance and 20 min of resistive (strength) training components. In addition, we conduct telephone follow-ups to assist in surmounting barriers to exercise. Tables 1 and 2 include the gait/balance training plan and resistance exercise program description.

**Table 1:** Gait/balance training plan

Exercise	Time	Description
Walking forward and backward	30 s × 2	Two individuals stand facing each other, holding hands. As one person walks backward, the other walks forward.
Walking side to side head motion	30 s × 2	Stand straight, and walk forward, with the eyes focused straight ahead, turning the head from side to side every 5 steps, keeping a straight course and avoiding drifting.
Walking up/down head motion	30 s × 2	Stand straight, and walk forward, eyes straight ahead, and nod the head up and down approximately every 5 steps.
Static standing	1 min each	Performed on both a firm and thick foam surface. Stand with eyes open, shifting weight from right to left leg. Repeat with eyes closed.
Standing partial tandem	1 min each	Performed on both a firm and thick foam surface with eyes open and closed.
Tandem standing heel to toe	1 min each	Performed on both a firm and thick foam surface with eyes open and closed.
Standing with head turns	1 min each	Performed on both a firm and thick foam surface with anterior, posterior, and lateral foot positions.
Single leg stance	30 s each	Performed on firm surface, raise right leg, balancing as long as possible. Repeat with other leg.
March in place	30 s × 2	Performed on both a firm and thick foam surface, march in place slowly.

**Table 2:** Resistance exercise program description

Resistance exercise	Muscle targeted	Exercise description
Calf raises	Gastrocnemius and soleus	Stand with their feet shoulder width apart and their knees fully extended and will push up on their toes as high as possible, keeping the torso erect.
Lunges	Gluteus maximus, hamstrings, quadriceps, and gastrocnemius	Standing with feet shoulder width apart, and hands on the hips. Take one step directly forward, keeping torso erect, and back knee slightly bent. With lead leg planted firmly in front, allow the lead hip and knee to slowly flex. Continue to flex until the trailing knee is only a few inches from the floor. Alternate legs and repeat.
Supine leg curls	Hamstring muscle group	Lie on the floor with legs bent to a 90° angle. Resistance band tubing is placed underneath the feet while the handles are placed one in each hand. Slowly, bring both heels away from the body until both legs are fully extended. Once fully extended, bring both feet back up toward the body until back in starting position.
Supine leg extensions	Abdominal and hip flexor muscles	Lying on the floor with legs bent to a 90° angle, feet flat on the floor. The resistance band tubing is placed under the foot, and the band handles with palms facing in. Extend the working knee from 90° until the leg is straightened. Slowly bring the extended leg back to the starting position. Repeat the set with the opposing leg.

#### Attention control protocol

Participants in the attention control group receive a journal to record their clinic appointments and standardized breast cancer survivorship education based upon information from the American Cancer Society. At each data collection encounter, the intervention research assistant discusses the information in each pamphlet, allowing time for questions related to the material. In addition, participants in the attention control group receive telephone calls every other week which entail a social visit and reminder of data collection/attention intervention appointments to equalize contact further.

### Criteria for discontinuing or modifying allocated interventions

Participants can leave the study for any reason if they wish to do so without consequences. The investigator can also end participation if the participant does not attend study visits. The patient data collected up to that moment will be included in the analysis.

### Strategies to improve adherence to interventions

The following strategies are used to increase adherence to the exercise program. (1) The exercise program is designed to be done at home, moving away from supervised sessions, to be more in context with natural living conditions; (2) the exercise program components are designed to be completed either in one session or split sessions; (3) the exercise program was designed to minimize injuries/soreness by beginning light-moderate and increasing over time; (4) the exercise program contains a self-monitoring Exercise Diary in REDCap and weekly telephone follow-ups to provide coaching regarding exercise-related issues and encourage completion of the Exercise Diary throughout the study period.

### Relevant concomitant care permitted or prohibited during the trial

Participants in the attention control group agree not to begin a new exercise program or change their level of exercise during the study.

### Outcomes

The primary outcome is to assess the effects of exercise on gait, balance, and lower extremity muscle strength in 312 women randomized to either a 16-week home-based gait/balance intervention or an attention control educational cancer survivorship condition. The gait assessment includes spatiotemporal metrics (cadence, speed, foot strike angle, stance percent, stride length, and swing percent) and lower extremity joint kinematics (hip, knee, and ankle joint angles). The APDM Opal (Mobility lab v1, APDM, Inc., Portland, OR) inertial measurement units (IMU) system assesses gait. The APDM IMUs are wireless sensors for measuring motion that allows clinicians to perform gait assessments quickly and straightforwardly [17, 18]. Balance is assessed using the Sensory Organization Test (SOT) performed using a NeuroCom Balance Master (NeuroCom International Inc, Clackamas, OR) and the EquitTest System (v8.0). The SOT measures the participants’ ability to effectively use visual, vestibular, and somatosensory information to maintain balance. The SOT equilibrium score has demonstrated moderate to excellent test-retest reliability in healthy older adults and individuals with Parkinson’s disease [19–21]. Isometric strength of hip, knee, and ankle flexor and extensor muscles is assessed using an isokinetic dynamometer, Biodex BX Advantage (Biodex Medical Systems, Shirley, NY, USA), and the computer software program version 3.29 and 3.30 [22–25]. We also assess neuropathy symptoms using the FACT-Taxane Additional Concerns Subscale and nerve conduction velocity of the sural and peroneal nerve action potentials [26, 27]. Different outcomes will be analyzed separately. Multiple comparison methods such as the Bonferroni-Holm Method will be applied to control family-wise error rates.

### Participant timeline

Table 3 shows the participant timeline.

**Table 3:** Participant timeline

Recruitment Screening Study information	
**Baseline visit:** Informed consent Baseline assessment of gait, balance, muscle strength Neuropathy symptoms patient-reported questionnaire Nerve conduction velocity assessment Randomization	
**Home-Based Physical Activity Intervention group**	**Educational attention control group**
Week 1–week 4	Week 1–week 16
Gait/balance exercise and resistance exercises 3 days per week Exercise diary Follow-up phone call weekly	Standardized breast cancer survivorship education from American Cancer Society Follow-up phone call bi-weekly
Week 5–week 16 Gait/balance exercise and resistance exercises 3 days per week Exercise diary Follow-up phone call bi-weekly	
**End of treatment visit:** Baseline assessment of gait, balance, muscle strength Neuropathy symptoms patient-reported questionnaire Nerve conduction velocity assessment	**End of treatment visit:** Baseline assessment of gait, balance, muscle strength Neuropathy symptoms patient-reported questionnaire Nerve conduction velocity assessment

### Sample size

Power analyses were performed through a Monte Carlo simulation approach with the software Mplus [28, 29]. Observations were spaced at 0 and 16 weeks, with the number of weeks since baseline as the time metric to evaluate the efficacy of the 16-week intervention.

#### SEM approach

The variance population parameters recommended by Muthén and Muthén were used (intercept variance = 0.50, slope variances (intervention = 0.10, intercept and slope covariances = 0.00), and residual variance at all waves = 0.50). Homogeneity of variances was assumed between the treatment and control groups. To reflect effective randomization of participants to conditions, we modeled no mean difference between treatment and control conditions at baseline. The differences in slopes between the treatment and control conditions during the intervention period are the focal parameter to be adequately powered. Given alpha = .05, a two-tailed hypothesis test, and the view that a power value of .80 will be adequate to detect a treatment effect, a minimum sample of *N* = 312 participants (based on recruitment of 2 or more participants per week for 3 years) with 20% attrition and 10% periodic non-response, is needed.

#### Intention to treat (ITT) approach

A full-information maximum likelihood approach for an intent-to-treat analysis is used, and a Monte Carlo simulation with 10,000 replications suggests we will be able to detect a minimum standardized effect of 0.30 with a probability of correctly rejecting a false null (power) of .81. If the recruitment rate is closer to 3 per week resulting in a sample of *N* = 468, the minimum detectable standardized effect is 0.25. By including additional control variables (all ES’s = .10), the minimum detectable effect sizes decrease to 0.27 and 0.22, respectively. A recent and relevant meta-analysis reported effect sizes for exercise intervention effects on similar outcomes ranging from ES = 0.30 to ES = 0.84 [30]. We will compare the mean differences between groups and the rates of change (slopes) between the groups.

### Assignment of interventions: allocation and blinding

Randomization to study groups is achieved using a computer-generated random numbers list (REDCap software, Nashville, TN) by the study statistician. A sealed, consecutively numbered, opaque envelope containing the subject’s group assignment (Exercise Intervention or Educational Attention Control) is opened to reveal the participant’s study group assignment. The study statistician generates the allocation sequence. The project manager enrolls participants, and the research assistant assigns participants to intervention groups. It is not an open-label study. The PI, data collectors, and the principal statistician are blinded. The data collector is blinded to the study group assignment. There is no unblinding procedure. No blocking or stratification is used.

## Data collection, management, and analysis

### Assessment and collection of outcomes

The assessment of gait, balance, and lower extremity muscle strength is performed by personnel with Master of Science in Kinesiology trained to perform these assessments. In addition, the assessment of neuropathy symptoms patient-reported questionnaire and the nerve conduction velocity are performed by a member of the research team and a neurologist, respectively. Outcomes are collected at baseline and at 16 weeks to provide baseline and end of intervention data. These data collection points reflect current knowledge concerning the impact of exercise on gait, balance, and lower extremity muscle strength from pre- to post-intervention [31–34]. The outcomes assessment occurs at the Human Functional Performance Laboratory at the University of South Florida.

### Assessment of gait

**Fig. 3:**
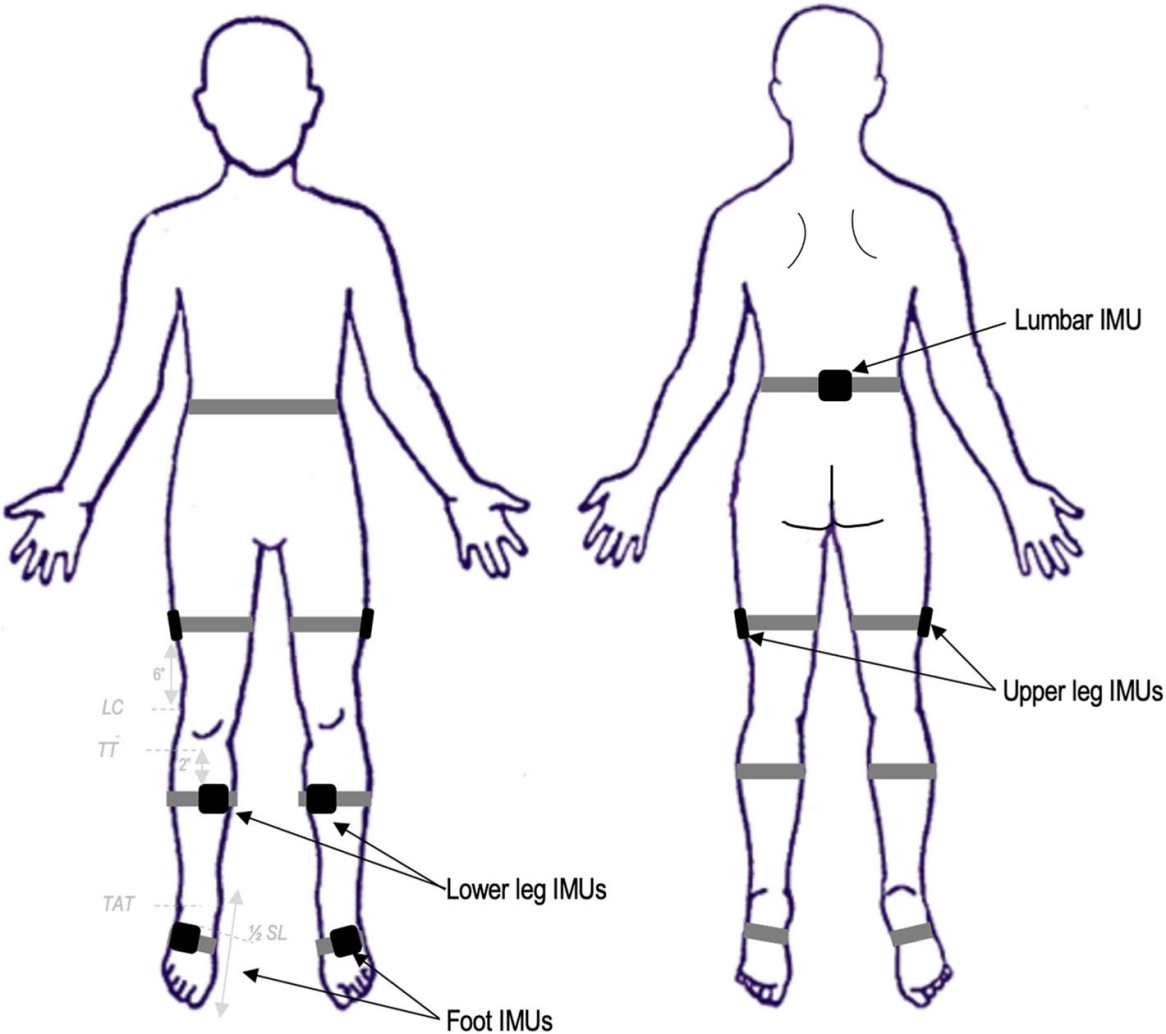
APDM Opal Sensors Placement. IMUs (inertial measurement units), LC (lateral condyle), TT (tibial tuberosity), TAT (tibialis anterior tendon), SL (shoe length)

The APDM Opal (Mobility lab v1, APDM, Inc., Portland, OR) inertial measurement units (IMU) are used to assess gait. The APDM IMUs are wireless sensors for measuring motion that allows clinicians to perform gait assessments quickly [17, 18]. The APDM Opal wireless sensors are positioned directly on the participant’s skin at the sacrum, laterally on each upper and lower leg, and each foot (Fig. 3). The wireless sensors are located at specific body landmarks. As shown in Fig. 3, the lumbar sensor is centered on the low back, at the base of the spine, and between the right and left posterior-superior iliac spines. The upper legs’ sensors are placed on the side of the thigh, midline, and 6 in above the femoral condyles. The lower leg’s sensors are located 2 in below and medial to the tibial tuberosity. The top medial corner of each foot sensor is placed at the intersection of the anterior tibialis tendon and the half-length of the shoe. Each of the seven sensors is secured with double-sided skin tape to limit displacement. IMUs are configured for synchronized logging at 128 Hz.

Before starting the walking assessment, participants are instructed to assume a neutral standing posture for 10 s and walk for approximately 2 min. Next, participants are instructed: “When ready, stand against this wall and place your feet in line with the tape arrows. Ensure your heels, back, and head are resting against the wall with your hands resting on your sides. Hold this position for about 10 seconds. Then, begin walking at the usual, comfortable walking speed. Next, turn and continue walking at a normal pace back and forth for 2 minutes. I will say STOP when the test is complete, then please take a seat.” During the testing, participants are instructed to “keep walking at your normal pace.” As shown in Table 4, the spatiotemporal metrics of gait (*r* > 0.86; ICC > 0.90) and gait kinematics (*r* > 0.7; ICC > 0.75) measures used in this study demonstrated excellent and good test-retest reliability, respectively [35].

**Table 4:** Reliability of outcome measures

Reliability		*r*	ICC
Isometric strength	Hip extension (Nm)	0.74	0.85
	Hip flexion (Nm)	0.92	0.96
	Knee extension (Nm)	0.94	0.96
	Knee flexion (Nm)	0.95	0.97
	Ankle plantarflexion (Nm)	0.92	0.94
	Ankle dorsiflexion (Nm)	0.91	0.96
Gait			
Spatiotemporal metrics	Cadence (steps/min)	0.97	0.98
	Gait speed (m/s)	0.86	0.93
	Foot strike angle (degrees)	0.93	0.96
	Stance (%)	0.97	0.99
	Stride length (m)	0.95	0.98
	Swing (%)	0.97	0.99
Kinematics	Hip flexion (degrees)	0.70	0.82
	Hip extension (degrees)	0.70	0.82
	Knee flexion (degrees)	0.71	0.77
	Knee extension (degrees)	0.82	0.86
	Ankle plantarflexion (degrees)	0.94	0.95
	Ankle dorsiflexion (degrees)	0.93	0.93
Balance	Composite equilibrium score (%)	0.87	0.67

*r* Pearson’s correlation, *ICC* intraclass correlation coefficient

The assessment of gait includes six spatiotemporal parameters: cadence [steps/min], speed [m/s], foot strike angle [degrees], stance percent [% of gait cycle time GCT], stride length [m], and swing percent [% of GCT]. In addition, the assessment of gait includes the sagittal plane kinematics (range of motion ROM) of three lower extremity joints: hip flexion-extension ROM [degrees], knee flexion-extension ROM [degrees], and ankle dorsiflexion-plantarflexion ROM [degrees]. Preprocessing of raw data and extraction of spatiotemporal gait variables and joint ROM are performed using APDM’s mobility lab software (Version 1). The mobility lab software computes the mean and standard deviation for each gait outcome variable from approximately 50–60 gait cycles collected during gait assessment. After testing, the mobility lab software automatically generates a report in CSV format containing the mean and standard deviation for each outcome variable. The CSV files are then imported to the REDCap database for further statistical analysis.

### Assessment of balance

Balance is assessed using the Sensory Organization Test (SOT) performed using a dynamic posturographic EquiTest® System (NeuroCom® International, Inc., Clackamas, OR, USA). The SOT measures the participants’ ability to effectively use visual, vestibular, and somatosensory information to maintain balance. Before testing, participants are strapped into a harness attached to a support beam and positioned on the fixed platform in the proper foot alignment. There are six different conditions for the SOT: (1) eyes open with a fixed floor; (2) eyes closed with a fixed floor; (3) eyes open, fixed surroundings, and floor sways; (4) eyes closed, fixed surroundings and floor sways; (5) eyes open, surroundings sway and fixed floor; (6) eyes open, both surroundings and floor sway. Participants are asked to stand as still and stable as possible during each test, and each testing condition is measured three times. The support surface that participants stand on and the visual surroundings move or sway in response to a participants’ sway during the test. The SOT provides a composite equilibrium score that reflects the participant's sway in both anterior and posterior directions. High scores indicate greater stability and less sway.

The NeuroCom Balance Manager Software (NeuroCom® International, Inc., Clackamas, OR, Unites States) is used to process the raw data and calculate the balance outcome variable, the SOT composite equilibrium score. The SOT equilibrium score has demonstrated moderate to excellent test-retest reliability in healthy older adults and individuals with Parkinson’s disease [19–21]. As shown in Table 4, the study protocol for assessing the SOT composite equilibrium scores demonstrated moderate test-retest reliability (*r* > 0.87; ICC > 0.67) [35].

### Assessment of lower extremity muscle strength

Isometric strength of hip, knee, and ankle flexor and extensor muscles is assessed using an isokinetic dynamometer, Biodex BX Advantage (Biodex Medical Systems, Shirley, NY, USA), and the computer software program version 3.29 and 3.30. The test protocols have been established based on the Biodex BX Advantage manual and recommendations from other studies [22–25]. The dominant and non-dominant legs are tested. Before each test, participants will become familiar with the procedures by performing 2–3 contractions as warm-ups. During the test, participants are guided with standardized instructions during the test to encourage sub-maximal muscle performance. Participants are stabilized in the chair with shoulder and abdominal straps. The anatomical axis of rotation is aligned to the dynamometer axis using visual inspection and manual palpation. The isometric tests include three sets of sub-maximal muscle contractions, each set lasting 5 s separated by 10 s rest intervals. The isometric strength of the hip muscles is assessed with subjects in a supine position with the hip joint positioned at 45° of flexion [22]. For assessment of the knee muscles, subjects are positioned in sited position with the knee positioned at 60° of flexion [36, 37]. To evaluate the ankle muscles, subjects are positioned in a sited position with the ankle joint positioned in a neutral position (0°) [38]. Participants performed three sets of isometric contractions for extensor and flexor muscles of the hip, knee, and ankle joints. The reliability of muscle strength values measured using isokinetic dynamometers is excellent [39, 40]. It has shown good to excellent reliability in healthy older adults and patients with hereditary motor sensory neuropathy [25, 41]. As shown in Table 3, the lower extremity muscle strength measures used in this study demonstrated good to excellent test-retest reliability (*r* > 0.7; ICC > 0.80) [35].

The Biodex Advantage BX 4. X Software is used to calculate the peak torque [Newton-meters, Nm] (i.e., highest force output during each sub-maximal muscle isometric contraction) and the average peak torque (i.e., average force output of a given set). The average peak torque (Newton-meters, Nm) obtained in each series is used for data analysis.

### Assessment of neuropathy symptoms

Patient-reported symptoms are assessed using the Functional Assessment of Cancer Therapy-Taxane (FACT-Taxane) Additional Concerns subscale. FACT-Taxane contains five domains comprised of questions about physical well-being, social well-being, emotional well-being, functional well-being, and additional concerns subscale. The FACT-Taxane Additional Concerns subscale comprises sixteen questions that address symptoms specific to neuropathy, with scores for each question ranging from 0 (not at all) to 4 (very much). Higher scores indicate more neuropathy symptoms [26].

### Nerve conduction velocity

A combination of a clinician-based test and a patient-reported questionnaire has been suggested as a proper assessment tool to evaluate taxane-induced neuropathy [27]. Sensory and motor nerve conduction studies are conducted on the sural and peroneal nerves. The nerve conduction velocities and amplitudes of each nerve are collected for comparison over time [27].

### Plans to promote participant retention and complete follow-up

Follow-up telephone calls to study participants in the intervention group are made weekly for the first month, then bi-monthly. Participants in the attention control group receive monthly phone calls as social contact and serve as a reminder for data collection appointments and review educational brochure topics. In addition, four newsletters are sent monthly to all study participants. Newsletters contain study recruitment updates and general health tips that do not influence study outcomes.

### Data management

A codebook for all study variables is created. A trained research assistant enters data into SPSS, verified weekly. Data cleaning uses frequency counts for all variables at each data collection point to check for outliers. All possible strategies are employed to prevent missing data, including in-person data collection by trained research assistants. Missing data is considered analytically through full information maximum likelihood (FIML) procedures available in standard software for generalized mixed to reduce potential bias and loss of power that would otherwise occur when using traditional listwise deletion for missing data [42]. The codebook does not include paper case report forms (CRFs). Instead, we use the HIPPA-Compliant REDCap database as the electronic data capture for the study. We export the REDCap data or CSV files to be analyzed using SPSS and R for data analysis.

### Confidentiality

Participants’ data is stored using a participant identification number at the screening time. According to research guidelines, the key to the identification code list is only available to the research team during the study and documented and safeguarded by the principal investigator. Only personnel directly related to the project or the University human subject oversight office has access to the data. All data will be reported as group data.

### Data analysis

Univariate descriptive statistics will be used to describe the characteristics of the sample, gait, balance, and lower extremity muscle strength. Values for the exercise will be divided into quartiles to determine the uppermost and lowest values to compare with published literature [43]. Analysis of univariate statistics allows us to assess the normality of distributions for our continuously distributed variables, patterns of missing values, and whether univariate outliers may be present that could distort subsequent bivariate and multivariate analyses. By implementing appropriate link functions in the generalized linear model framework, distributional assumptions, such as normality and potential non-linearity, will be addressed. Potential non-normality may also be addressed by implementing robust estimation through Huber-White standard errors (i.e., “sandwich” estimators), also implementable in the generalized mixed model framework. We do not aggregate outcomes. Instead, we are looking at a specific change of each metric between baseline and 16 weeks and comparing study groups. In addition, we will perform baseline comparisons between groups. Any unbalance covariate(s) will be adjusted in multivariate analyses. We do not plan any subgroup analyses.

### Monitoring

#### Data monitoring and trial steering committee

An independent Data and Safety Monitoring Board (DSMB) is available to monitor the trial’s progress and the participant’s safety. The board will include a biostatistician, an oncologist, and a senior oncology nurse faculty member not directly associated with the study. At quarterly intervals throughout the project, the board receives a comprehensive report of data regarding (1) all causes of mortality and (2) morbidity (hospitalizations, emergency room visits, and injuries/problems resulting from exercises, such as delayed-onset muscle soreness (DOMS), any reports of falls or injuries resulting from exercise, the development of lymphedema in previously unaffected participants, and safety concerns associated with exercise. Additionally, the rate of recruitment refusal and subject attrition will be tracked and reported in these quarterly reviews. These parameters are differentially monitored between the intervention and control groups.

If members of the research team identify concerns or problems, they will be reported to the University’s Institutional Review Board (IRB) and NIH as indicated by the IRB. Annual progress reports to the IRB and NIH will include a summary of the DSMB’s activity findings and any adverse events regarding human subjects.

#### Adverse event reporting

The risk to participants in this study is expected to be minimal. However, there is a small risk of falls in participants who experience gait and balance difficulties due to neuropathy resulting from taxane therapy. The study team advises safety precautions such as having a sturdy chair nearby and a person supervising the exercise to increase safety and minimize the risk of falls. Participants randomized to the exercise intervention may experience DOMS due to increased tension on muscle fibers during the first few exercise sessions. Exercise-related DOMS decreases with repeated stimuli and does not typically require medical attention. In addition, a breach of confidentiality is a potential risk. Study materials have unique study numbers and are kept secured in locked files to minimize the risk of a confidentiality breach.

Patients are interviewed at each visit for assessment and asked about any adverse events. Surveillance for adverse events (AEs) and other relevant clinical circumstances associated with study participation occur at in-person visits scheduled at 0 and 16 weeks using a standardized Adverse Event Record Form. Adverse event reporting is unmasked to the study statistician and Data Safety and Monitoring Board (DSMB). Any AE will be investigated immediately, assessed for relatedness and expectedness by the study PI, and reported in a timely fashion as required by the DSMB and University of South Florida IRB.

### Ethics and dissemination

#### Ethics approval and consent to participate

This study has been approved by the University of South Florida Institutional Review Board (Pro00040035). The consent process takes place at the USF Human Functional Performance Laboratory. After discussing the study and answering any questions, a trained research assistant obtains written consent from participants. All signed consent forms are kept in a locked file in the principal investigator’s office. The study’s ethical approval is included in Additional File 2. The study’s informed consent will be available upon reasonable request. This trial does not involve collecting biological specimens for storage.

#### Protocol amendments

Any significant modifications to the protocol that may impact the conduct of the study, the potential benefit of the participant, or affect participant safety will require a formal amendment to the protocol. Amendments to the protocol will need approval from the USF IRB before implementation.

## Discussion

The proposed study examines the effectiveness of gait/balance training plus resistance exercises on gait, balance, and lower extremity muscle strength in individuals who have completed treatment with taxane-based chemotherapy. Declines in peripheral nerve function secondary to neurotoxic chemotherapy have been well documented. At present, no interventions have been demonstrated to prevent or alleviate taxane-induced peripheral neuropathy. Research focusing on designing and implementing gait/balance training and resistance exercises for individuals receiving chemotherapy is necessary for clinicians to prevent debilitating sensory and motor neuropathy in their patients, thus preserving physical function. Ameliorating the physical dysfunction and pain associated with CIPN using convenient, home-based cost-effective, and feasible interventions are urgently needed. This clinical trial will directly impact rehabilitation strategies to improve the quality of life in this patient population.

## Trial status

This study protocol version number is 2, dated March 12, 2022. The recruitment of participants started in August 2020. Currently, there are 33 participants recruited (17 in the intervention group and 16 in the control group). Recruitment is estimated to be completed in July 2024.

## Supplementary Information

**Additional file 1.** SPIRIT 2013 Checklist.

**Additional file 2.** Ethical Approval.

## Data Availability

The datasets used and analyzed during the current study are available from the corresponding author on reasonable request.

## Abbreviations

AEs: Adverse events
B-HAPI: Home-Based Physical Activity Intervention
CIPN: Chemotherapy-induced peripheral neuropathy
DSMB: Data Safety and Monitoring Board
FIML: Full information maximum likelihood
HFPL: Human Functional Performance Laboratory
ICC: Intraclass correlation coefficient
IMUs: Inertial measurement units
ITT: Intention to treat
RCT: Randomized clinical trial
SOT: Sensory Organization Test
VAS: Visual analog scale
