# Supplementary material for: Assessing gait, balance, and muscle strength among breast cancer survivors with chemotherapy-induced peripheral neuropathy (CIPN): study protocol for a randomized controlled clinical trial
Source: Trials. 2022 Apr 27;23:363. doi: 10.1186/s13063-022-06294-w (PMC9044705; doi:10.1186/s13063-022-06294-w)
Supplement: Supplementary file 2 — Additional file 2. Ethical Approval. [file 13063_2022_6294_MOESM2_ESM.pdf]

UNIVERSITY OF SOUTH FLORIDA
USF RESEARCH & INNOVATION

RESEARCH INTEGRITY & COMPLIANCE
Institutional Review Boards, FWA No. 00001669
12901 Bruce B. Downs Blvd, MDC35, Tampa, FL 33612-4799
(813) 974-5638 FAX (813) 974-7091

9/30/2019

Constance Visovsky, Ph.D.
College of Nursing
12901 Bruce B. Downs Blvd. MDC22
Tampa, FL 33612

RE: **Expedited Approval for Initial Review**
IRB#: Pro00040035
Title: Home-Based Physical Activity Intervention for Persistent Taxane-Induced Neuropathy

Study Approval Period: 9/30/2019

Dear Dr. Visovsky:

On 9/30/2019, the Institutional Review Board (IRB) reviewed and **APPROVED** the above application and all documents contained within, including those outlined below.

Approved Item(s):
Protocol Document(s):

Visovsky Protocol V1 09-26-2019 Clean.docx

Consent/Assent Document(s)*:

Visovsky Consent V1 09-19-2019 Clean.docx.pdf

*Please use only the official IRB stamped informed consent/assent document(s) found under the "Attachments" tab. Please note, these consent/assent documents are valid until the consent document is amended and approved.

It was the determination of the IRB that your study qualified for expedited review which includes activities that: (1) present no more than minimal risk to human subjects, and (2) involve only procedures listed in one or more of the categories outlined below. The IRB may review research through the expedited review procedure authorized by 45 CFR 46.110 and 21 CFR 56.110. The research proposed in this study is categorized under the following expedited review category:

(4) Collection of data through noninvasive procedures (not involving general anesthesia or sedation) routinely employed in clinical practice, excluding procedures involving x-rays or microwaves. Where medical devices are employed, they must be cleared/approved for marketing.

(6) Collection of data from voice, video, digital, or image recordings made for research purposes.

(7) Research on individual or group characteristics or behavior (including, but not limited to, research on perception, cognition, motivation, identity, language, communication, cultural beliefs or practices, and social behavior) or research employing survey, interview, oral history, focus group, program evaluation, human factors evaluation, or quality assurance methodologies.

Your study qualifies for a waiver of the requirement for signed authorization as outlined in the HIPAA Privacy Rule regulations at 45 CFR 164.512(i), which states that an IRB may approve a waiver or alteration of the authorization requirement provided that the following criteria are met (1) the PHI use or disclosure involves no more than a minimal risk to the privacy of individuals; (2) the research could not practicably be conducted without the requested waiver or alteration; and (3) the research could not practicably be conducted without access to and use of the PHI. An alteration of HIPAA Authorization is granted for the phone screen portion of the study only, exempting the study team from the Privacy Rule’s requirement that subjects sign and date the Authorization during screening.

As the principal investigator of this study, it is your responsibility to conduct this study in accordance with IRB policies and procedures and as approved by the IRB. Any changes to the approved research must be submitted to the IRB via an Amendment for review and approval. Additionally, all unanticipated problems must be reported to the USF IRB within five (5) business days.

We appreciate your dedication to the ethical conduct of human subjects research at the University of South Florida and your continued commitment to human research protections. If you have any questions regarding this matter, please call 813-974-5638.

Sincerely,

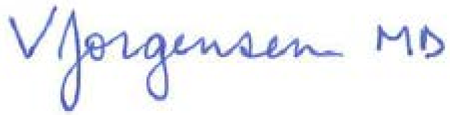

E. Verena Jorgensen, M.D., Chairperson
USF Institutional Review Board
